# Sepsis‐induced systemic inflammation and tau pathology examined in the PS19 transgenic mouse model

**DOI:** 10.1002/alz70855_107400

**Published:** 2025-12-25

**Authors:** Quan Vo, Dina Nacionales, Kristi Biswas, Carmelina Gorski, Lyle L Moldawer, Philip A Efron, Paramita Chakrabarty

**Affiliations:** ^1^ University of Florida, Gainesville, FL, USA; ^2^ University of Florida College of Medicine, Gainesville, FL, USA

## Abstract

**Background:**

Inflammation is a complex biological process that has been shown to be a critical component of the neurodegenerative process in Alzheimer's disease (AD). While intracerebral LPS alters tau pathology in AD mouse models, whether chronic inflammatory signaling from the periphery could alter brain‐resident tau protein remains to be determined. To answer this question, we induced intra‐abdominal sepsis in the transgenic PS19 mice which express human P301S mutant tau.

**Method:**

PS19 mice were subjected to cecal ligation and puncture combined with daily chronic stress at 6‐months of age, the age that coincides with emerging pathology in the brainstem and spinal cord. Mice were aged to either 8‐months (‘prodromal cohort’) or onset of bilateral hindlimb paralysis (‘survival cohort’). Brain and spleen tissue were collected for immunohistochemical analysis and flow cytometry to assess phosphorylated tau (AT8) and immune alterations. Comparisons were made with sex‐matched naïve control animals.

**Results:**

In the prodromal septic cohort, splenic flow cytometry revealed persistent immune alterations, with septic females exhibiting reduced CD8^+^ T‐cell indicating continuing lymphopenia. Septic mice of both sexes had increased levels of myeloid‐derived suppressor cells indicating immune suppression. Notwithstanding high levels of inter‐sample variability, our data indicates that systemic inflammation did not accelerate AT8‐positive tau pathology in male or female septic mice. In the survival cohort, we observed sex differences in lifespan. While septic males exhibited a higher median age of survival (422d vs. 304d in naïve males), septic females displayed an accelerated time to paralysis (324d vs. 393 days in naïve females). The splenic immune cell distribution mostly returned to baseline at this time, though the septic mice had lower spleen weights indicating continuing impaired immune function. In the end‐stage mice that received no treatment, male PS19 mice had higher AT8‐tau burden in the hippocampus than females. Surprisingly, male septic mice showed lower AT8‐ptau in the hippocampus, while the cortex and brainstem showed equivalent levels between septic and control mice.

**Conclusion:**

Our findings suggest that systemic inflammation could affect lifespan and regional AT8‐tau burden based on sex. Further experiments are warranted to identify cell‐type specific changes to identify mechanistic links between systemic inflammation and AD‐type tau pathology.

